# Quality of Care in Contraceptive Services Provided to Young People in Two Ugandan Districts: A Simulated Client Study

**DOI:** 10.1371/journal.pone.0027908

**Published:** 2011-11-21

**Authors:** Gorrette Nalwadda, Nazarius M. Tumwesigye, Elisabeth Faxelid, Josaphat Byamugisha, Florence Mirembe

**Affiliations:** 1 Department of Nursing, School of Health Sciences, Makerere University College of Health Sciences, Kampala, Uganda; 2 Department of Epidemiology and Biostatistics, School of Public Health, Makerere University College Health Sciences, Kampala, Uganda; 3 Division of Global Health, Department of Public Health Sciences, Karolinska Institutet, Stockholm, Sweden; 4 Department of Obstetrics and Gynecology, School of Medicine, Makerere University College of Health Sciences, Kampala, Uganda; University of Cape Town, South Africa

## Abstract

**Background:**

Low and inconsistent use of contraceptives by young people contributes to unintended pregnancies. This study assessed quality of contraceptive services for young people aged 15–24 in two rural districts in Uganda.

**Methods:**

Five female and two male simulated clients (SCs) interacted with 128 providers at public, private not-for-profit (PNFP), and private for profit (PFP) health facilities. After consultations, SCs were interviewed using a structured questionnaire. Six aspects of quality of care (client's needs, choice of contraceptive methods, information given to users, client-provider interpersonal relations, constellation of services, and continuity mechanisms) were assessed. Descriptive statistics and factor analysis were performed.

**Results:**

Means and categorized quality scores for all aspects of quality were low in both public and private facilities. The lowest quality scores were observed in PFP, and medium scores in PNFP facilities. The choice of contraceptive methods and interpersonal relations quality scores were slightly higher in public facilities. Needs assessment scores were highest in PNFP facilities. All facilities were classified as having low scores for appropriate constellation of services. Information given to users was suboptimal and providers promoted specific contraceptive methods. Minority of providers offered preferred method of choice and showed respect for privacy.

**Conclusions:**

The quality of contraceptive services provided to young people was low. Concurrent quality improvements and strengthening of health systems are needed.

## Introduction

Providing quality contraceptive care for sexually active young people is a concern in many countries [1]. The bio-social gap, the period between menarche and marriage, has widened [2]. This has dramatically increased the time period during which young people need contraceptives. The high rates of teenage pregnancy (25%) and unintended pregnancy (46%) [3], often lead to unsafely performed abortions, which account for nearly half of all maternal deaths among young women in Uganda [4]. If all unintended births were eliminated in Uganda, it is believed that the total fertility rate would drop from 6.7 to 5.1 children per woman [5].

The demand for contraceptives among Ugandan young people is 45 percent and 57 percent for age groups 15–19 and 20–24 respectively [6]. However, only 11 percent and 21 percent use contraceptives in these respective age groups [3]. Reducing the unmet need for contraceptives is a critical goal for the health service in Uganda [7]. Despite an apparent high level of awareness of contraceptive methods, the level of use has remained low and inconsistent among young people [3]. The low quality of contraceptive services may be one of the many contributing factors to non-use of contraceptives among young people.

While use of modern contraceptives in Uganda is only 18 percent among all married women, more than half (58%) of these users discontinue use within 12 months of initiation [3]. The discontinuation rates for the three main methods are estimated to be 61 percent for oral pills, 46 percent for progestin-only injections and 71 percent for condoms [3]. The reasons for such high discontinuation rates are not clearly understood. Evidence from other low resource settings has linked poor quality of services to high rates of discontinuation, reduced utilization, non-compliance and hence high unintended fertility [8].

The current Ugandan national health policy seeks to increase access to quality, affordable, acceptable and sustainable contraceptive services [7], but provision of quality contraceptive services for young people remains under debate. A recent qualitative study recounted changing perceptions and behavioral shift towards contraceptive use among young people, but noted service level barriers for those who had overcome all other obstacles and tried to access contraceptive services [9]. Earlier studies among providers in Uganda have identified health system factors that deter contraceptive use to include: shortage of skilled providers, limited supplies and equipment, poor service organization, and provider imposed restrictions [10], [11]. While these studies imply barriers to quality services, they did not focus on measuring quality. Other studies have been limited to specific project areas or assessed only a few aspects of quality, so further research has been recommended [12], [13]. Demographic health surveys have no or only modest information on facility operations, infrastructures and providers' behavior. Furthermore, no study in Uganda has examined the quality of contraceptive services that young people receive. The aim of this study was therefore to assess and analyze the quality of contraceptive services provided to young people aged 15–24 in order to identify areas for improvement.

The study was guided by a theoretical framework of assessing quality of care in contraceptives services proposed by Bruce and Jain [14]. This framework proposes six aspects of assessing quality, which are technical competences of providers, information given to users, choice of contraceptive methods provided, interpersonal relations, continuity mechanisms, and appropriate constellation of services, sometimes referred to as appropriateness and acceptability. The six aspects reflect attributes of the services clients experience as critical for contraceptive adoption and continued utilization [15]. Studies that have used this framework have shown that quality of care can impinge on individuals' decisions to use or not use modern methods and also the choice of methods [15], [16]. It is not clear if all the aspects of quality care are relevant constituents of service quality in Africa. No study has used the framework to assess the quality of services provided to young people or has determined the relative importance of the different aspects.

## Materials and Methods

### Study setting

A facility-based cross-sectional study was conducted between September 2009 and April 2010 in two rural districts in Uganda: Mityana and Mubende. The two districts have a total population of nearly 1 million people [17], of which 60 percent is less than 18 years of age [18]. Both districts have teenage pregnancy rates of 30 percent compared to the average of 26 percent in rural areas in general and 20 percent in their urban counterparts [3]. Each district has three health sub-districts. A health sub-district covers a catchment population of approximately 100,000 people. In each district the health sub-district with a hospital was purposively selected as study site to ensure representation of a cross-section of all health care facilities. There are three types of health facilities: public, private not-for-profit (PNFP), and private for profit (PFP). Contraceptive services are provided in all these facilities. Public and PNFP service delivery points included hospitals and health centers while PFP were comprised of private clinics, pharmacies, and drug stores. Both pharmacies and drug stores dispense or sell contraceptives and other drugs, except that pharmacies are of a larger scale. Contraceptive delivery points were identified using the recent Ministry of Health lists for public and PNFP facilities, while information about PFP facilities was obtained from National Drug Authority lists and private clinics registry. All lists were obtained at two district health offices. These lists comprised 35 public, 11 PNFP and 84 PFP facilities (20 clinics, 4 pharmacies, and 60 drug stores). Twenty-one of the facilities were registered as drug stores but operating as clinics. All facilities were included in the study.

### Study design

The simulated client method was employed to assess the quality of contraceptive services provided to young people. This method was preferred in order to decrease the level of intrusiveness that is caused by the presence of an independent observer during a consultation. In addition, the method reduces faulty recall, and captures both the observable and intangible aspects of the care-giving process [19]. Each simulated client (a data collector posing as a client) visited a specified service delivery point only once.

### Data collection procedures and tools

Two male and five female simulated clients (SCs), aged 15–24, were recruited from the two districts through youth leaders. The SCs were either graduate midwives or had advanced secondary education. They had good communication skills and were fluent in both English and the local language of the study area (Luganda). This enhanced the SCs ability to collect and report reliable information from their observations and interactions with the providers. The SCs were trained by the first author and a sociologist for three days using six case scenarios and role-plays. The scenarios represent the main contraceptive methods and were used as a guide to elicit guidance, information, and services from providers (appendix S1). Each female SC was given one while each male SC was given two case scenarios to perform throughout the data collection. SCs were systematically assigned to facilities. In facilities where the contraceptive method in the case scenario was not offered, the case scenario was replaced with another. The condom case scenario did not yield much information and was used less frequently since some providers tended to just give or sell the condoms to the SCs without much of interaction and information. The first author and the field research staff assisted the SCs in locating the service delivery points. Money was given to the SCs to pay for contraceptive commodities, consultation fees, and transport.

The SCs visited the health care providers and requested contraceptive services. The health care providers were not aware that these particular clients were involved in research. The SCs interactions with the providers were mainly in the local language. Immediately after the encounter, the SC narrated his/her interaction with the health care provider to the first author. Thereafter, the SC was interviewed either by the first author or by two trained interviewers (nurse/midwives) on the events during their visit using a structured questionnaire. The scenarios and questionnaire were designed by the research team, pilot tested in Kampala District, and modified before use to ensure that they captured information on all quality of care variables. The scenarios and the questionnaire were designed to include indicators for the six aspects of assessing quality according to the Bruce-Jain framework as described in literature [8], [14], [20], [21]. However, in our study the element of technical competence was partially assessed through needs assessment. The questionnaire comprised of 61 questions that assessed the six aspects of quality of care as perceived by the client (client's needs, choice of contraceptive methods, information given to users, client-provider interpersonal relations, constellation of services, and continuity mechanisms). The response to each question was “Yes” or “No”. Questions were worded in a neutral sense to avoid a biased response. Data on health care provider and facility background characteristics were also collected. Furthermore, data from SCs narrative accounts, and questions related to waiting time, consulting time, provider bias, access, costs of contraceptive services and client satisfaction were also collected, although these results are reported in another paper.

### Data management and analysis

The data from the completed questionnaires was entered into a computer using EPIDATA V.3 and was later exported to STATA V.11 for cleaning and analysis. The unit of analysis was the health facility. The responses of SCs to quality of care related questions were coded 0 for “No” and 1 for “Yes”. A “Yes” was equivalent to a unit score on any variable in the six aspects of assessing quality. A frequency distribution of the quality assessment variables was carried out. Exploratory factor analysis was also performed for data reduction and to create composite quality scores [22]. Before applying factor analysis all variables were screened for correlation with other variables. A correlation command in STATA helped generate tables of inter-variable correlation coefficients for each aspect of quality. From these tables variables that did not have a correlation coefficient of at least 0.2 with at least one other variable were eliminated. This reduced the number of variables to 50. Thereafter factor analysis was performed using the principal component analysis method. This resulted in different factor loadings, which showed the variation that the different families of variables had in each aspect of quality. Six individual factor analyses were performed according to aspects of assessing quality (needs assessment, choice of contraceptive methods, information given to users, interpersonal relations, constellation of services, and continuity mechanisms). Two factors, which contributed to 50–75 percent of the variability in each aspect of quality, were retained [22].

A varimax rotation procedure that modifies the factor analysis results to get a clearer pattern was applied [23]. Quality assessment variables with factor loadings of 0.3 or greater were included [23], while variables having the largest contribution to the variation outside the two factors were eliminated. This reduced the number of variables to 44. Factor analysis was run again for the remaining variables in each aspect and for the two retained factors. Table 1 shows rotated factor loadings for each aspect of quality arranged in two retained factors explaining most of the variance in the data. The intention of the factor analysis was not to classify the factors (1 and 2); nonetheless, the factor loadings showed the relative importance of each variable. Table 1 also shows how the remaining variables faired in terms of variability in the final model. The variables in each of the six aspects of assessing quality listed in Table 1 ultimately defined quality. In addition, eliminated variables are shown. From this stage a predict command of STATA V.11 was applied to generate a score, which was categorized into high, medium and low quality. The predict command collapses variables to get an average score from influences of all the variables remaining in the model [23]. All variables loaded strongly on factor 1. Variables that loaded strongly on factor 2 also had a smaller factor loading on factor 1, although this is not shown in Table 1. Thus, only the first factor loading contributed to the computation of the scores. It is this new categorized variable that was used to assess the quality of the different service providers.

**Table 1:** Rotated factor loadings of aspects of quality of care in contraceptive services.

Quality of care variables†	Factor 1	Factor 2	Uniqueness
***Needs assessment (N = 104)***			
Provider obtained menstrual and contraceptive history		.78	.38
Asked whether client wanted to conceive a child	.89		.20
Asked how long client wanted to wait before next birth	.89		.20
Asked about previous contraceptive use experiences		.79	.36
*Eigenvalue*	*1.60*	*1.24*	
*Variance explained*	*40.0*	*31.1*	
***Information given to users (N = 122)***			
Client told about a variety of methods		.71	.49
Client told who can/not use various contraceptive methods		.62	.54
Shown or told how adopted method works	.47		.61
Told how to use the method adopted	.48		.62
Warned of potential side effects	.49		.53
Instructed on how to handle problems	.76		.42
Informed of warning signs	.60		.58
Client given written or pictorial information on methods	.51		.66
Informed of methods that protect against STIs		.66	.55
Provider told client about the benefits of method adopted		.43	.74
Told where to go in case of complications	.76		.40
*Eigenvalue*	*3.5*	*1.3*	
*Variance explained*	*31.8*	*11.6*	
***Choice of contraceptive methods (N = 108)***			
Provider asked which method is preferred by client		.82	.31
Provider told client about short acting methods	.79		.35
Provider told client about long acting methods	.76		.40
Client asked to choose a method		.82	.32
Preferred method was available		.38	.81
Told about method-specific side effects	.59		.52
Told of the option to switch methods	.65		.57
Client given appropriate referral when method of choice not available	.56		.65
Told the number of contraceptive methods available	.67		.34
*Eigenvalue*	*3.20*	*1.51*	
*Variance explained*	*35.4*	*16.8*	
***Interpersonal relations N = 125***			
Provider welcomed client		.75	.42
Client given adequate answers to all questions	.80		.32
Provider explained what was going to be done to obtain client's consent	.47		.70
Provider treated the client in a friendly manner		.46	.72
Client shown respect for privacy		.65	.56
Client received care in a clean environment		.69	.50
Client felt received satisfactory care	.85		.24
Provider asked whether client understood	.56		.67
Based on service received client would come back to this provider	.75		.33
*Eigenvalue*	*2.81*	*1.67*	
*Variance explained*	*31.3*	*18.6*	
***Constellation of services N = 125***			
Provider screened clients for contraindications -blood pressure and weight		.82	.31
Provider asked about illness client might have had before		.66	.55
Provider advised client about STIs		.73	.45
Provider advised client about dual method use	.81		.32
Client told about integrated services (STIs/HIV, ANC, MCH, Postnatal)	.83		.31
Client told about youth center available in the area-sexuality	.76		.41
*Eigenvalue*	*2.0*	*1.63*	
*Variance explained*	*33.2*	*27.2*	
***Continuity mechanisms (N = 126)***			
Client told to return if she/he had doubts	.84		.29
Provider scheduled a follow-up visit	.78		.38
Client informed of alternative sources of care		.68	.42
Client left provider feeling like consultation will be kept confidential	.31		.90
Provider recorded client's visit in clinic book or client card		.85	.25
*Eigenvalue*	*1.62*	*1.10*	
*Variance explained*	*32.5*	*22.1*	
***Overall quality (N = 116)***			
*Eigenvalue*	*6.6*	*3.0*	
*Variance explained*	*43.7*	*19.7*	

† Excluded statements; **Needs assessment**-“wanted to perform pelvic exam”; **Information given to users**- none; **Choice of contraceptive method**- “Client given method of choice (where applicable)”, “Provider told other method besides the one adopted”, “Received information without any single method being promoted by provider”; **Interpersonal relations-** “Nobody else could hear during client-provider consultation”, “Provider permitted client to ask questions”, “Nobody else was in the room/space during client- provider consultation”, “Provider said some things a client did not understand”, “Door closed or curtain drawn when client with provider”, “Provider did nothing to breach clients' privacy/confidentiality”, “Client given IEC material”, “Provider raised her/his voice or shouted at client”, “Provider made comment about client's age or appearance”; **Constellation of services**- “Contraceptive posters, job aids observed in service area”, “Provider advised about HIV testing”; **Continuity mechanism**- “Client given appointment card with follow up”, “Client told about availability of community distribution for refill”.

Thereafter, chi-square test or Fishers' exact test were used to assess relationships between the categorized quality scores and health facility type. In addition, overall quality (which included all variables in the six aspects) was computed following similar steps, and in this factor analysis the number of variables was reduced to 28. Mean scores were also computed according to the six aspects using the retained variables to summarize the data describing quality of care. To test the differences between these mean scores of quality by facility type, we performed 1-way analyses of variance. We also applied Kruskal-Wallis test, a non-parametric version of one-way-analysis of variance based on ranks which can deal better with small numbers in some cells. To check for internal consistency, Cronbach's alpha was calculated for each of the six aspects of measuring quality. A p-value of 0.05 and less was considered to indicate significant statistical difference. The level of confidence interval was 95%.

### Ethics statement

Research ethics committees of Makerere University, Uganda National Council for Science and Technology, and the Regional Ethics committee in Stockholm, Sweden approved the study. After explaining the purpose of the study, informed consent was obtained from service providers six months before the study period when we conducted data collection for another study in the same area. Providers were informed that SCs would visit their clinics in the next couple of months seeking contraceptive services and agreed to participate. Safety issues to SCs were addressed in the pre-training. Written informed consent was also secured from all SCs.

## Results

### Background characteristics

A total of 128 SC visits out of the 130 contraceptive delivery points were analyzed. These included 34 public (27%), 10 PNFP (8%), and 84 PFP (65%) health facilities. Simulated clients were attended to by midwives during most visits (67%), followed by nursing assistants (13%), medical doctors (10%), clinical officers (8%) and non-medical staff (2%). Most of the contraceptive service providers were women (74%). Male SCs made 18 (14%) visits, while female SCs made 110 (86%) visits. The different case scenarios used according to facilities are shown in Table 2.

**Table 2:** Simulated client and service provider background characteristics by facility type.

	Facility type				
Background characteristics	*Total*	*Public*	*PNFP*	*PFP*	*P-*
	*n (%)*	*n (%)*	*n (%)*	*n (%)*	*Value*
*Case scenario-contraceptive method option*					
FAM case (21 female, 4 Male SC visits)	25 (19.5)	2 (5.8)	5 (50.0)	18 (21.4)	
Pill case (all female SC visits)	28 (21.8)	5 (14.7)	1(10.0)	22 (26.2)	
Oral pill side effects case (Female SC)	27 (21.1)	6 (17.6)	2 (20.0)	19 (22.6)	—
Injection case ( 24 female, 4 male SC visits)	28 (21.8)	9 (26.5)	0 (0.0)	19 (22.6)	
Implant case (10 female, 5 male SC visits)	15 (11.7)	11 (32.3)	2 (20.0)	2 (2.4)	
Condom case (all male SC visits)	5 (3.9)	1 (2.9))	0 (0.0)	4 (4.7)	
*Sex of simulated clients*					
Female	110 (85.9)	28 (82.3)	8 (80.0)	74 (88.1)	*0.51*
Male	18 (14.1)	6 (17.6)	2 (20.0)	10 (11.9)	
*Sex of service provider*					
Male	33 (25.8)	4 (11.7)	2 (20.0)	27 (32.1)	*0.06*
Female	95 (74.2)	30 (88.2)	8 (80.0)	57 (67.8)	

*P-values based on Fisher's exact test, —number too small in some cells, SC- simulated client, FAM- fertility awareness methods.*

### Description of quality variables

Selected quality assessment variables including some outliers eliminated during factor analysis are described below in each aspect of quality. Health care providers attending to the SCs discussed fertility intentions in 17 percent of the encounters while information on how the adopted method works was given during 45 percent of the visits. In 27 percent of the encounters, the SCs were instructed on how to handle possible problems during use of contraceptives. The majority of providers (64%) promoted a specific contraceptive method. To assist clients in choosing a contraceptive method, most providers (86%) told the clients at least one additional method besides the one chosen. The SCs were offered their method of choice in only 31 percent of visits. For interpersonal relations, almost all providers (96 percent) permitted SCs to ask questions, and 67 percent of the providers gave adequate answers according to the clients. The SCs felt their privacy was respected in 42 percent of the encounters. A few providers (4%) raised their voice or shouted at clients during the consultations. In terms of constellation of services, a minority of providers advised clients about STIs/HIV (18%) and dual method use (12%), and only seven percent of the providers gave information about other relevant sexual and reproductive health services. To facilitate continuity, instructions about return visits to the facility in case of doubts were given in 42 percent of the visits while 28 percent of the clients left the facility with a scheduled follow up visit, and 12 percent of the providers recorded the current visits.

### Quality of care based on mean scores

The raw mean scores of quality (based on retained items shown in Table 1 for the six aspects of assessing quality) were mostly low. On a score range from 0–9, the mean scores were 5.3 (CI 4.8–5.7) for choice of contraceptive method and 5.1 (CI 4.7–5.5) for interpersonal relations. In the remaining aspects of assessing quality, the mean scores were 4.9 (CI 4.4–5.4) for information given to users (range 0–11), 0.4 (CI 0.2–0.5) for constellation of services (range 0–6), 2.1 (CI 1.9–2.3) for continuity mechanisms (range 0–5), and 1.0 (CI 0.8–1.2) for needs assessment (range 0–4). The mean overall score of quality for the six elements was 14.2 (CI 13.1–15.4) of the possible 28. Although there were no statistically significant differences by facility type, public facilities had slightly higher means than PNFP and PFP facilities (Table 3).

**Table 3:** Mean scores of aspects of quality in contraceptive services by facility type.

	Facility type					
Aspects of quality (score range)	*Total*	*Public*	*PNFP*	*PFP*	*ANOVA*	*p-*
	*Mean (SD)*	*Mean (SD)*	*Mean (SD)*	*Mean (SD)*	*F-value*	*value*
Needs assessment (0–4)	1.1 (1.1)	1.5 (1.2)	1.4 (1.4)	0.9 (0.9)	3.4	0.24
Information given to users(0–11)	4.9 (2.8)	6.1 (2.9)	5.1 (1.8)	4.4 (2.7)	4.7	0.37
Choice of contraceptive method (0–9)	5.3 (2.5)	6.39 (2.3)	5.8 (2.1)	4.8 (2.5)	5.0	0.76
Interpersonal relations (0–9)	5.1 (2.1)	5.7 (2.1)	6.1 (1.3)	4.8 (2.2)	3.1	0.28
Constellation of services (0–6)	0.4 (1.1)	0.4 (0.8)	0.4 (1.0)	0.3 (0.7)	0.2	0.41
Continuity mechanisms (0–5)	2.1 (1.2)	2.6 (1.3)	2.4 (1.0)	1.9 (1.2)	4.4	0.67
Overall quality (0–28)	14.2 (6.3)	17.1(6.0)	16.8 (3.2)	12.9(6.1)	6.1	0.25

*SD- standard deviation, N varied, Needs assessment (N = 104), Information received (N = 122), Choice of contraceptive method (N = 118), Interpersonal relations (N = 125), Constellation of service (N = 125), continuity of care (N = 126), overall quality (N = 116). ANOVA- Analysis of variance, Overall quality score is based on 28 items with sufficient factor loading.*

The results from ranked scores based on Kruskal-Wallis test showed significant differences by facility type for information received (p<0.01), method choice and continuity of care (p<0.001), interpersonal relations (p = 0.04), but there was no difference for constellation of service (p = 0.49) and needs assessment (p = 0.07). Overall quality was also statistically different by facility type (p<0.001).

The internal consistency for each of the elements of quality of care was tested with Cronbach's alpha. It was estimated to be 0.50 for needs assessment, 0.78 for information given to users, 0.74 for choice of contraceptive method, 0.70 for interpersonal relations, 0.60 for constellation services, and 0.50 for continuity mechanisms, while for the overall quality it was 0.88.

### Quality of care based on factor analysis scores

Results from cross-tabulation to assess relationships between the categorized quality scores and health facility type showed low quality in all of the six aspects assessed (Table 4). The quality scores differed significantly by facility type in five of the six aspects (needs assessment, information given to users, choice of contraceptive method, continuity mechanisms, and interpersonal relations). Private for profit facilities had relatively low scores in all aspects of quality. The results indicate that PNFP facilities generally had medium quality scores in relation to information given to users, choice of contraceptive methods and continuity mechanisms. The choice of contraceptive methods and interpersonal relations scores were slightly higher in public compared to PNFP and PFP facilities. Constellation of services had the weakest scores of all the six aspects of assessing quality. All facilities were classified as having low scores for appropriate constellation of services. Needs assessment scores were highest in PNFP facilities. Overall quality (composite quality score for the six aspects) was rather low, and differed by facility type (p<0.05). The overall quality scores differed significantly between public and PFP facilities.

**Table 4:** Quality of care scores in contraceptive services received by facility type.

	Facility type				
Aspects of quality	*Total*	*Public*	*PNFP*	*PFP*	*Chi-sq.*
	*n (%)*	*n (%)*	*n (%)*	*n (%)*	
*Needs assessment (N = 104)*					
Low	42 (40.4)	12 (41.4)	1 (14.6)	29 (42.7)	
Medium	37 (35.6)	6 (20.7)	2 (28.3)	29 (42.7)	
High	25 (24.0)	11 (37.9)	4 (57.1)	10 (14.7)	***
*Information given to users (N = 122)*					
Low	41 (33.6)	8 (23.5)	1 (12.5)	32 (40.0)	
Medium	41 (33.6)	10 (29.4)	6 (75.0)	25 (31.2)	**
High	40 (32.8)	16 (47.1)	1 (12.5)	23 (28.8)	
*Choice of contraceptive method (N = 118)*					
Low	41(34.7)	5 (15.2)	1 (16.6)	35 (44.3)	
Medium	38 (32.2)	11 (33.3)	4 (66.6)	23 (29.1)	***
High	39 (33.1)	17 (51.5)	1 (16.6)	21 (26.6)	
*Interpersonal relations ( N = 125)*					
Low	42 (33.6)	8 (25.0)	2 (22.2)	32 (38.1)	
Medium	42 (33.6)	7 (21.9)	3 (33.3)	32 (38.1)	**
High	41 (32.8)	16 (53.1)	4 (44.4)	20 (23.8)	
*Constellation of services (N = 125)*					
Low	100(80.0)	25 (75.8)	7 (77.8)	68 (81.9)	
High	25 (20.0)	8(24.2)	2 (22.2)	15(18.1)	
*Continuity mechanisms (N = 126)*					
Low	47 (37.3)	12 (35.3)	0 (0.0)	35(42.2)	
Medium	38 (30.2)	9 (26.5)	6(66.7)	23 (27.1)	**
High	41 (32.5)	13 (38.2)	3 (33.3)	25 (30.1)	
*Overall quality (N = 114)*					
Low	39 (33.6)	6 (18.7)	1 (16.7)	32 (41.0)	
Medium	39 (33.6)	10 (31.2)	3(50.0)	26 (33.3)	**
High	38 (32.7)	16 (50.0)	2 (33.3)	20 (25.6)	

*Quality of care scores categorized as low, medium, and high tertiles based on factor analysis, N = refers to number of SC visits, N varied by aspect of quality due to missing variables in some cases where “No” or “Yes” was not applicable,*

**p<0.05,

***p<0.01, p-value- Fishers exact test. There was no score for moderate category in constellation of services.

### *Relationship between quality of care and sex of providers and simulated clients*

The overall quality of care differed significantly according to the sex of the SC (p<0.05) with female SCs receiving slightly better quality than their male counterparts. Sex of the providers had no effect on the quality of care. There were no significant differences in quality scores by sex of provider (p = 0.7).

## Discussion

The study ultimately assessed aspects of quality contraceptive care in order to make recommendations for improvement. The results of this study suggest that the quality of contraceptive services provided to Ugandan young people is low. Both means and categorized quality scores were low for all aspects of quality assessed. This low quality of services might be suggestive of difficulties young people experience in receiving modern contraceptives, as well as gaps in existing services. It has been articulated that high-quality contraceptive services could reduce maternal morbidity and mortality as well as poverty and therefore promote sustainable development [5]. We speculate that provision of quality contraceptive services would increase contraceptive use, consistent with other studies that have related quality of care to contraceptive use [8], [15]. The limited contraceptive use in Uganda [3], may be partly attributable to low quality services in addition to other factors, such as difficulty in access to services, weak health systems, and cultural beliefs [9]. One plausible explanation for the low quality established in our study may be limited capacity of the health care providers to give information and services based on up to date scientific evidence. This might also limit them in offering information to the clients. A quality improvement intervention study with a training component targeting private practicing midwives revealed improved counseling and technical skills among participants after the intervention [13].

Our results further indicate that information and instructions given to clients were incomplete, inaccurate, and usually unclear. Although the providers mentioned the different contraceptive methods, they generally gave suboptimal information to users and frequently recommended specific methods to clients. This implies that it was often not possible for clients to make their own informed choice. Comprehensive and correct information enhances informed choice, a key feature in widening clients' knowledge and dispelling myths about contraception [24]. The PNFP and PFP health facilities in particular gave moderate or minimal information. This suggests that providers may have limited competence in explaining and providing methods, or have limited time to convey sufficient information to clients. In addition, providers might have negative attitudes and also a lack of interest and desire to give contraceptive information to young people. Similar findings were reported in previous studies [11], [25].

The quality related to choice of contraceptive method was slightly higher compared to other aspects of quality in facilities visited by the SCs. Nevertheless, there was a violation of personal choice in some cases by providers not offering clients the opportunity to choose their preferred method. Being able to select the preferred method is an important component of quality contraceptive services that could increase young clients' acceptance and continued utilization of the chosen method [24]. Given the insufficient supplies of contraceptives at the facilities [11], allowing a woman to access and choose from multiple contraceptive methods would increase the likelihood that at least one method of choice would be available. Receiving a preferred contraceptive type increases acceptance and continued use, but also increases the prospect of switching to other methods [26]. Thus, health care providers should be aware that young clients have the right to be informed about a variety of contraceptive methods, access to them, and the right to choose their preferred method. However, providers can only meet client needs optimally if provided with updated information, appropriate contraceptive mix and proper infrastructures [27].

This study provides evidence of suboptimal interaction between young people and providers, which is an important aspect of quality of contraceptive services. More than two thirds of the client-provider interactions were rated to be of low or medium quality. This may indicate negative health care provider attitudes related to young people's contraceptive use, which has also been observed in a previous qualitative study conducted in Uganda [28]. Improving client provider interaction translates into clients' decision making about methods, their participation in the care, and better outcomes such as satisfaction. Previous research has also shown that interpersonal relations are related to contraceptive use [29].

Surprisingly, the constellation of services was the weakest aspect in the quality of care for contraceptive services. Integration or service mix including dual method use, which is a strong component of appropriate constellation of services, was noted to be limited in the facilities. Information about STIs, HIV, precautions for contraceptive use, and dual method use which are important for the youth was infrequently given. These findings reflect weak constellation of services, consistent with results from a previous study from Uganda [12].

Data from the current study indicate low quality regarding continuity mechanisms. Few health care providers informed clients about time for revisits, and clients were rarely informed about sources for re-supplies. Given the importance of continued use and compliance with methods, it is imperative that providers discuss and agree on criteria for follow up visits with their clients.

Our study therefore provides relevant information on health sector performance with regards to contraceptive services for young people. The quality indicators point toward limited compliance by providers with quality of care norms in contraceptive services. Our results indicate that the capacity of facilities to provide quality contraceptive services varies between public, PNFP and PFP. Overall, the quality of care was low but also differed by facility type. Slightly higher total quality indices were noted among public facilities compared with private-sector facilities. The scale of operation of some PFP health care facilities such as drug stores is relatively small, impeding privacy and time available for clients. It is worth noting that in a minority of the encounters, services were provided by non-medical staff, who may be family members of the health care providers. This may greatly influence the quality of services offered. Thus, stressing the quality in both private and public sector providers might boost on service outcomes [30]. While the sex of the providers was not related to levels of quality, the observed differences by sex of the SC may imply that male clients face more difficulties when seeking contraceptive services.

To improve the quality of contraceptive services to young people, the authors suggest that remedial efforts need to deepen stewardship for quality of care in the health agenda, and support training and integration of quality contraceptive services in all sectors at all levels. The results indicate a need to reinforce better interpersonal communications between provider and clients, provision of correct information, and broadening of contraceptive method choices in order to increase the use of contraceptives. In addition, continuous quality improvement strategies in contraceptive services are required to increase uptake and build demand for contraceptives among young people. Innovative approaches to concurrently address health systems and quality issues are required to improve contraceptive use by young people. This hopefully will bridge the unmet need for contraception and reduce unintended fertility and its consequences.

Our study was limited by the number of facilities, thus our results should be interpreted in light of the small sample size. The study relied on SCs for evaluation of quality of services provided. It is possible that SCs may not have accurately recalled all the information, but to overcome this, the interview was carried out immediately after the encounter with the provider [19]. In addition, evaluation was strengthened by the selection of competent SCs with good communication skills, use of more than one SC in the study and by each SC presenting only one case scenario. The SCs were also trained carefully before data collection started. Variation in sex of SCs and of case scenarios is a potential source of fault but applying factor analysis eliminated inconsistent variables and cases. Some of the PFP facilities were drug stores with limited services but these were assessed based on methods they provided according to the case scenario. Use of Yes and No responses (scale 0–1) has limitations of lack of variability. Cronbach's alpha appears to be slightly low (<0.7) for three aspects of quality (needs assessment, continuity of care and constellation of services), possibly because each had fewer or less related questions. However, the several ways used in analyzing the data including frequency distribution, Kruskal-Wallis test, summative score, and scores created by factor analysis is considered strength.

### Conclusion

Overall, the quality of care in contraceptive services provided to young people was low, with significant gaps requiring improvement. Significant shortfalls were observed in assessing clients' needs, and measures for continuity and proper constellation of services. Whereas some providers listed the methods, clients made their choices based on limited information. Our study showed comparable levels of poor quality of contraceptive services offered to young people in both public and private sector facilities. Both mean and categorized quality scores were slightly higher in choice of contraceptive method and interpersonal relations.

## Supporting Information

Appendix S1 **The six case scenarios used by simulated clients.**
(DOC)
