# Supplementary material for: Quality of Care in Contraceptive Services Provided to Young People in Two Ugandan Districts: A Simulated Client Study
Source: PLoS One. 2011 Nov 21;6(11):e27908. doi: 10.1371/journal.pone.0027908 (PMC3221686; doi:10.1371/journal.pone.0027908)
Supplement: Appendix S1 — The six case scenarios used by simulated clients. (DOC) [file pone.0027908.s001.doc]

**Appendix S**1: The six case scenarios used by simulated clients

| **Case 1: Injectable (DMPA)**  A married young woman or man aged 20 years with a two-year-old child. She/ his wife have never used contraceptives before but want to control fertility because her husband/he has so many other children. She/ his wife has never had any pelvic examination before and is on her 3^rd^ day of the menstrual period. | **Case 2: Oral contraceptive**  A young-looking 16-year-old schoolgirl, having unprotected sex with a steady boyfriend and has no children. She is not sure if she is pregnant or not. She asks the provider for assistance in choosing an oral contraceptive method. She heard from friends that they can prevent pregnancy, nothing more. Have no health conditions (for example, diabetes or high blood pressure). |
| --- | --- |
| **Case 3: Implant**  A 23-year-old married woman or man, seeking recommendations for a contraceptive method. She/ he has two children (aged four and two) and does not wish to have any other child in the next five years. She/ he knows little about the methods but heard of a method where something can be inserted under the woman’s skin and wants to try it. She/ he has not used contraceptives before, but has unprotected sex. | **Case 4: Oral contraceptive side effects**  A young woman aged 17-19 years is using pills and does not like it any more. She asks the provider whether there is anything she can do about the nausea she has been experiencing since she started taking the pill two months ago. She likes the convenience of the pill—it does not interfere with the spontaneity of sex and it is more effective than condoms, which her boyfriend has been using. |
| **Case 5: Condom**  A young man is visiting his grandparents and he found a girl he is attracted to in the village, and wants to have sexual intercourse for the first time. He is so scared of making her pregnant and getting HIV. He heard about some contraceptive methods that can be used by a man, and would like to be assisted in learning about them. | **Case 6: Fertility awareness method (FAM)**  A married woman or man aged 20 years with two children, one three years and the other one year old. She/he has never used contraceptives, doesn’t want to have other children soon, but wants to have unprotected sex. The profile requires the client to reject all methods offered except FAM. The client is afraid that the partner may not allow the use of other methods. |
